# Assessing the feasibility and acceptability of a financial versus behavioural incentive-based intervention for community health workers in rural Indonesia

**DOI:** 10.1186/s40814-021-00871-7

**Published:** 2021-06-23

**Authors:** Thomas Gadsden, Stephen Jan, Sujarwoto Sujarwoto, Budiarto Eko Kusumo, Anna Palagyi

**Affiliations:** 1 The George Institute for Global Health, University of New South Wales, Sydney, Australia; 2 Faculty of Medicine and Health, The University of Sydney School of Public Health, Sydney, Australia; 3 Department of Public Administration, University of Brawijaya, Malang, Indonesia

**Keywords:** Community health workers, Indonesia, Incentives, Motivation, Performance

## Abstract

**Background:**

The World Health Organization recommends that community health workers (CHWs) receive a mix of financial and non-financial incentives, yet notes that there is limited evidence to support the use of one type of incentive (i.e. financial or non-financial) over another. In preparation for a larger scale trial, we investigated the acceptability and feasibility of two different forms of incentives for CHWs in Malang District, Indonesia.

**Methods:**

CHWs working on a cardiovascular disease (CVD) risk screening and management programme in two villages were assigned to receive either a financial or non-financial incentive for 6 months. In the financial incentives village, CHWs (n = 20) received 16,000 IDR (USD 1.1) per patient followed up or 500,000 IDR (USD 34.1) if they followed up 100% of their assigned high-risk CVD patients each month. In the non-financial incentive village, CHWs (n = 20) were eligible to receive a Quality Care Certificate for following up the highest number of high-risk CVD patients each month, awarded in a public ceremony. At the end of the 6-month intervention period, focus group discussions were conducted with CHWs and semi-structured interviews with programme administrators to investigate acceptability, facilitators and barriers to implementation and feasibility of the incentive models. Data on monthly CHW follow-up activity were analysed using descriptive statistics to assess the preliminary impact of each incentive on service delivery outcomes, and CHW motivation levels were assessed pre- and post-implementation.

**Results:**

Factors beyond the control of the study significantly interrupted the implementation of the financial incentive, particularly the threat of violence towards CHWs due to village government elections. Despite CHWs reporting that both the financial and non-financial incentives were acceptable, programme administrators questioned the sustainability of the non-financial incentive and reported CHWs were ambivalent towards them. CHW service delivery outcomes increased 17% for CHWs eligible for the non-financial incentive and 21% for CHWs eligible for the financial incentive. There was a statistically significant increase (p < 0.0001) in motivation scores for the performance domain in both villages.

**Conclusion:**

It was feasible to deliver both a performance-based financial and non-financial incentive to CHWs in Malang District, Indonesia, and both incentive types were acceptable to CHWs and programme administrators. Evidence of preliminary effectiveness also suggests that both the financial and non-financial incentives were associated with improved motivation and service delivery outcomes. These findings will inform the next phase of incentive design, in which incentive feasibility and preliminary effectiveness will need to be considered alongside their longer-term sustainability within the health system.

**Supplementary Information:**

The online version contains supplementary material available at 10.1186/s40814-021-00871-7.

## Key messages regarding feasibility

What uncertainties regarding feasibility existed prior to this study?

- The importance of providing community health workers (CHWs) a mix of financial and non-financial incentives is well recognised, yet there is insufficient context-specific evidence on which type of incentives are most effective and the feasibility of implementing such incentives within the health systems of resource-poor settings.

What are the key findings on feasibility from this study?

- Despite interruptions to implementation by unforeseen political and health system factors, both a performance-based financial incentive and a non-financial award were considered acceptable to CHWs and feasible by programme administrators, in the study setting of Malang, East Java, Indonesia. Additionally, both incentives showed evidence of preliminary effectiveness in terms of motivation and service delivery outcomes.

What are the implications of the findings on the design of the main study?

- In the next phase of incentive design, the feasibility of implementation and preliminary effectiveness will need to be considered alongside their longer-term sustainability within the health system. Our findings also reinforce the importance of articulating a theory of change and incorporating mechanisms to both assess and account for context (e.g. embedded process and/or realist evaluation) within the design of any subsequent larger scale trial.

## Introduction

The shortage of health workers in low- and middle-income countries (LMICs) is forecast to reach approximately 15 million by 2030, representing a major challenge to achieving universal health coverage (UHC) [1, 2]. In response, many LMICs have placed greater emphasis on the role of community health worker (CHW) programmes to expand coverage of primary health care services. CHWs are defined as members of the communities where they work, selected by and answerable to communities, supported by the health system but not necessarily a part of its organization, and with shorter training than professional workers [3]. CHWs may be salaried or volunteers but generally have less benefits compared to health workers who are formally recognised as part of the health system [4].

In the 40 years between the Alma Ata and Astana Declarations, a substantial body of evidence has emerged demonstrating the pivotal role of CHWs in improving access to essential health care services and health outcomes [5–7]. Knowledge on the factors that support CHW motivation and performance has also advanced. Recent evidence reviews identify a combination of technical enablers such as training, supervision and remuneration, and contextual factors including sustained political support and funding, community embeddedness and integration with the health system [8–11].

Incentives are a key component of CHW programmes that can reduce attrition and improve motivation and performance [8, 12]. However, there is a low certainty of evidence to recommend one type of incentive over another [13]. The 2018 World Health Organization (WHO) guidelines to optimise CHW programmes cite insufficient context-specific evidence as a key research gap and conclude that ‘evidence is not sufficiently granular to allow recommendation of specific forms of interventions, for example … which bundle of financial and non-financial incentives are most effective’ [14]. Recommendations to address this gap include evaluating CHW programmes from a broader lens to understand the role of contextual factors, such as community embeddedness, and their association with successful implementation [12, 13, 15].

Furthermore, the design of incentive schemes for health care providers and the mechanisms through which they influence behaviour remains poorly understood and fraught with unintended consequences [16]. The design of, and response to, appropriate incentives may be strongly influenced by the complex socio-cultural and political ecosystems within which CHW function, yet there is limited research testing this relationship [17]. This is particularly true for CHWs, who are often volunteers, and therefore may be driven by altruistic or reputational motives and therefore may not respond to extrinsic incentives as intended [18]. For example, Wagner et al. found that when CHWs in Uganda were incentivised to sell oral rehydration salts (ORS) to households and keep the revenue, it reduced their motivation and led to less household visits compared to CHWs providing ORS for free evidence [19]. Conversely, evidence from India commonly shows that CHWs focus their efforts on incentivised activities at the expense of other tasks [20, 21]. Such unintended effects could potentially be uncovered through initial proof of concept testing for acceptability and efficacy of incentives.

Further research is needed to enable health system planners to design feasible and effective incentives that support the retention and motivation of CHWs. This study investigated the feasibility of implementing and evaluating two different forms of incentives for CHWs in Indonesia, in preparation for a larger scale cluster randomised trial that aims to assess how the design of incentives influences the motivation of, and quality of care provided by, CHWs. The specific aims of this mixed-methods feasibility study were to explore the acceptability of incentive design, to identify refinements for more rigorous testing in a larger scale pragmatic randomised controlled trial and to conduct a preliminary assessment of impact.

## Methods

### Study setting

The study was undertaken in two rural villages in Malang District, East Java, Indonesia (see Table 1). Malang is the second largest district in East Java province with a population of 2,592,147 people distributed across 33 sub-districts and 390 villages [22]. Malang has 39 primary health centres or Pukesmas (1 per ~ 65,000 individuals) and 390 village health clinics or Ponkesdes (1 per ~ 7000 individuals) [22]. For pragmatic reasons, the two participating villages—Sepanjang Village and Kepanjen Village—were selected for the study based on their involvement in an ongoing programme of research on a mobile health-supported community-based intervention to optimise preventative care and treatment for cardiovascular disease (CVD) (SMART*health* [23]) and existing support from the heads of the village-level Puskesmas.

**Table 1:** Characteristics of the study villages in Malang District

Characteristics [22]	Kepanjen village	Sepanjang village
**Population**	107,955	85,546
**Infrastructure**	Higher resourced	Lower resourced
**Medical doctors in Pukesmas**	3	2
**Nurses and midwives**	3	2
**Community health workers (Kaders)**	18	20
**Accessibility from neighbourhoods**	Good	Poor
**High-risk CVD patients** [23]	587	566

### Participants

Kaders (the Indonesian community health workforce) are village-based volunteers who deliver community-based health and nutrition awareness, monitoring and screening activities for non-communicable diseases, maternal and child health, and immunisation [24]. Typically, these activities take place at monthly village health posts, known as Posbindu, which were developed specifically for the early detection, monitoring and follow-up of community members with non-communicable disease risk factors [25]. Outside of these events, Kaders visit households to follow up with families and provide additional services as needed. Kaders are required to attend a 3-day training course during which they learn the Posbindu curriculum, which includes health promotion, anthropometry and blood pressure and glucose measurement [25]. A village committee appoints Kaders from within their village, to which they are accountable. In Malang District, Kaders receive a monthly payment of 25,000 Indonesian Rupiah (IDR) (US$2). All Kaders involved in the delivery of the SMART*health* programme in the two participating villages (n = 40) were eligible to participate.

### Incentive design

The incentives to be tested were collaboratively designed by stakeholders from the District Health Authority, University of Brawijaya, and The George Institute for Global Health. Performance-based financial incentives are a common feature of CHW programmes and were deemed appropriate for this research by stakeholders [8]. The incentive value was established by taking into account recent incentive increases for CHWs in the district and the minimum wage policy for Malang District and East Java province. There is consistent evidence that public recognition is an important form of motivation for CHWs and our performance-contingent non-financial incentive was designed to leverage this effect [11].

### Intervention

Between February and July 2019, as part of the SMART*health* programme, Kaders were requested to follow up patients at high risk of CVD at least once per month to provide guidance on lifestyle modification, monitor and promote adherence to prescribed medicines and encourage medicines compliance (i.e. attendance of high-risk patients at nurse-led clinics to refill prescriptions). Follow-up visits were conducted in the patient’s home or during regular community gatherings for social or religious purposes. Typically, each Kader was assigned approximately 30 patients for follow-up.

Kaders in Sepanjang village (n = 20) were allocated to receive a monthly financial incentive based on the number of high-risk CVD patients they followed up. If Kaders followed up 100% of their assigned patients each month, they received 500,000 IDR (USD 34.1). If they did not achieve 100% follow-up, Kaders received 16,000 IDR (USD 1.1) per patient followed up. Furthermore, if a Kader attempted to follow up a patient three times without success, this was counted as a successful follow-up.

Financial incentives were distributed to Kaders by project supervisors in monthly supervision meetings. Kaders self-reported their performance in a monthly report, which documented their assigned patients, the date of follow-up of each patient and the results of the visit, including CVD monitoring data such as blood pressure, blood glucose levels and medication adherence. These were checked by project supervisors. During these meetings, supervisors and Kaders also discussed barriers to patient follow-up and potential solutions.

In Kepanjen village, Kaders (n = 20) were eligible to receive a non-financial incentive in the form of a Quality Care Certificate. The Kader with the highest number of patients followed up each month was awarded this certificate, which was co-signed by the District Health Authority and the village head. Kaders were presented the certificate by a representative from the village government at monthly facilitation meetings attended by village government members, village nurses, the SMART*health* team and occasionally representatives from the Puskesmas and District Health Office.

### Data collection and analysis

We used a pragmatic mixed-methods approach to data collection to maximise understanding of the feasibility and acceptability of the different inventive models, and their preliminary effectiveness. The four main data collection methods were as follows:

1. Focus group discussions with Kaders to understand their perceptions regarding the different forms of incentives. All Kaders in each village were invited to participate, and focus groups were conducted by the University of Brawijaya staff using a pre-defined discussion guide.
2. Semi-structured interviews with programme administrators to understand the contextual influencers (facilitators and barriers) of the intervention, acceptability of the incentive models and their perceived effectiveness. Interviews were conducted by the University of Brawijaya staff using a pre-defined questionnaire.
3. A pre- and post-implementation survey of Kaders using a structured instrument to determine the changes in motivation levels resulting during the incentivised time period.
4. Routinely collected data of service delivery outcomes (i.e. Kader follow-up activity levels) were analysed using descriptive statistics.

A focus group discussion guide and interview questions, developed in English and translated into Bahasa, were used to collect qualitative data. Focus group discussions and semi-structured interviews were conducted in the local language, audio-recorded, transcribed and translated into English. Qualitative data were analysed to explore the following themes: (1) barriers and facilitators to implementation of the incentive structure, (2) implementation progress and adaption required of intervention as needed and (3) contextual factors influencing success or failure of implementation.

The use of qualitative methods to measure motivation among CHWs has grown in the past decade [26–28]. We adapted an instrument developed by Prytherch et al. to measure Kader’s motivation before and after the intervention [29]. Our adaptation included a short demographic survey followed by 41 statements to which participants responded using a 4-point Likert scale with the following options: ‘strongly disagree’, ‘disagree’, ‘agree’ and ‘strongly agree’ (see Additional file 1 for the complete instrument). Kaders completed the survey in a group setting in each village prior to incentive implementation and again at the completion of the 6-month study period, with instructions provided by project staff.

Pre-post analysis of survey results was conducted using the Stata 16 software. The mean responses to the three survey domains (management, performance and individual) were compared and tested for any differences of significance using a paired t test; a mean score of 2.5 was considered the neutral midpoint on the 4-point scale, with a higher score reflecting a greater level of motivation. Unweighted means were used as all domains were afforded the same importance. Ten questions were negatively phrased and therefore reverse coded.

### Outcomes

The primary outcomes investigated were the feasibility and acceptability of the two different forms of incentives, including their design and method of administration. Secondary outcome measures were Kaders’ monthly patient follow-up activity and changes in motivation over the 6-month implementation period. Last, we considered what adaptations might be necessary for the practicality and implementation of a larger scale trial, based on the findings of this study.

### Ethics and consent

Implementation was formally discussed with the village head of each participating village prior to study commencement. Signed informed consent was obtained from all participants contributing data to the qualitative evaluation activities (focus groups and semi-structured interviews) and the motivational survey. Ethics approval was granted by the Human Research Ethics Committees of the University of New South Wales (HC190048) and the Medical Faculty of the University of Brawijaya (10/EC/KEPK/04/2018).

## Results

In total, 40 Kaders participated in the study: 20 in the financial incentive village (Sepanjang) and 20 in the non-financial incentive village (Kepanjen), all of whom were female. Kaders shared similar demographic characteristics in terms of age, years of education and time having worked as a Kader (see Table 2). Those in the financial incentive village were more likely to report having employment outside their role as a Kader (70%) compared to those in the non-financial incentive village (55%).

**Table 2:** Demographic characteristics of Kaders in the two study villages

Characteristic	Non-financial incentive (Kepanjen)	Financial incentive (Sepanjang)	Total
Female	20 (100%)	20 (100%)	40 (100%)
Mean age (years)	45	41	43
Age range	31–59	25–56	25–59
Mean number of years of schooling	13	11	12
Number of dependents (mean)	1.6	3	2.3
Additional employment	55%	70%	63%
Average number of years as Kader	14	12	13

There were 587 community members in the non-financial incentive village identified as being of high risk of CVD for follow-up by Kaders, equivalent to 33 patients for each Kader. In the financial incentive village, 566 village members were identified as high-risk patients for follow-up by Kaders, equivalent to 28 patients per Kader.

### Qualitative assessment of feasibility and acceptability

#### Focus group discussions

Thirteen Kaders from the non-financial incentive village (Kepanjen) and 12 from the financial incentive village (Sepanjang) participated in focus group discussions. Kaders in the financial incentive village reported that financial incentives were an important source of income for them: ‘yes (the incentive is important), in order to purchase gas for vehicles’ (age, 44; tenure, 4 years); ‘yes, it is important due to the fact that we need to build a saving’ (age, 36; tenure, 1 year); ‘It is important to provide aid for those who have financial issues’ (age, 49; tenure, 7 years). These Kaders also reported that health insurance and vouchers to purchase household goods may be desirable forms of non-financial incentives.

Kaders receiving the financial incentive also reported enjoying their work, and being motivated by their ability to help villagers, to work closely with Kader colleagues, to increase their own knowledge and to meet regularly with doctors and nurses. ‘I feel content as long as it benefits other people and my family in a positive aspect’ (age, 49; tenure, 7 years); ‘I am happy as this job benefits the society and the community’ (age, 33; tenure, 5 years); ‘I am happy because I can help other people’ (age, 36; tenure, 1 year).

Kaders in both study villages reported that they require some additional form of accreditation to legitimise their activities in the eyes of villagers. Kaders in the non-financial incentive village reported: ‘one of our issues is that we need a letter from the head of the village to support our activities, especially because we need to ask the patients to pay for the consumable health equipment we used. So far, the head of the village hasn’t issued that letter. We will be able to work better if we have that letter’ (age, 49; tenure, 5 years)*.*

Kaders in both villages also reported challenges in accessing male village members due to their work commitments, reluctance to visit monthly health promotion activities (Posbindu) and a general disregard towards the advice provided by Kaders.

#### Semi-structured interviews

Interviews with SMART*health* programme administrators (n = 2) provided qualitative evidence about the acceptability of the incentives. They reported that financial incentives are important motivators for Kaders, as most are economically disadvantaged. ‘Kader’s attitude towards financial incentives is very relevant. Because generally they have economic limitations. So, with the money incentive, Kaders who are generally housewives can be helped in carrying out follow-up. They do depend on incentive money’.

Both programme administrators considered the financial incentive amount to be appropriate: ‘Yes, it is appropriate. For Sepanjang [financial incentive] Kaders, the amount of incentives can support Kaders family for buying rice for a month’ (senior researcher, University of Brawijaya) The amount was also considered to align with recent incentive increases in the jurisdiction: ‘… in a number of cases in the Malang Regency area there has been an increase in incentive money in connection with each Kader formation. For example, Kader assigned to handle stunting were given 150,000 IDR. This increase is based on the discretion of the village head in allocating a portion of the village funds for the incentives of the Kader’ (SMARThealth programme coordinator)*.*

Programme administrators reported that there were mixed attitudes towards the non-financial incentive (Quality Care Certificate) among Kaders: ‘The attitude of Kepanjen [non-financial incentive] Kaders towards the Quality Care Certificate is that there is enthusiasm, but some are not enthusiastic. But in general, they feel happy doing activities as Kaders’ (SMARThealth programme coordinator). Additionally, one programme administrator questioned how effective the non-financial incentive will be in the long-term: ‘it is appropriate but for longer time I think it would be less effective since what Kaders actually need is recognition in the form of community supports. Say for example, some Kaders should walk quite far away to see high risk patients and she actually need someone who able to accompany her’ (senior researcher, University of Brawijaya)*.*

The criteria for awarding the Quality Care Certificate was reported to be appropriate: ‘This (will) indicate which Kaders are diligent and which are lazy. Based on healthy social competition, it is certainly appropriate if those given the certificate are those Kaders who are diligent and diligent in following up patients’ (senior researcher, University of Brawijaya)*.*

Both programme administrators reported that the most important motivating factors for Kaders were the opportunities for recognition and learning: ‘By becoming Kaders, they have benefited greatly. Widespread social interaction, understanding of health will be good, being able to differentiate licensed drugs from over-the-counter drugs, have skills in screening, and so on’ (SMARThealth programme coordinator). Furthermore, ‘the most important factor is not the certificate but some Kaders understand that by participating in Posbindu and following up high risk patients they will get other benefits not in terms of money but new knowledge and recognition from community members and of course she get free to have glucose test and get medication which sometimes also for her family members’ (senior researcher, University of Brawijaya).

### Barriers and facilitators to implementation

**Fig. 1:**
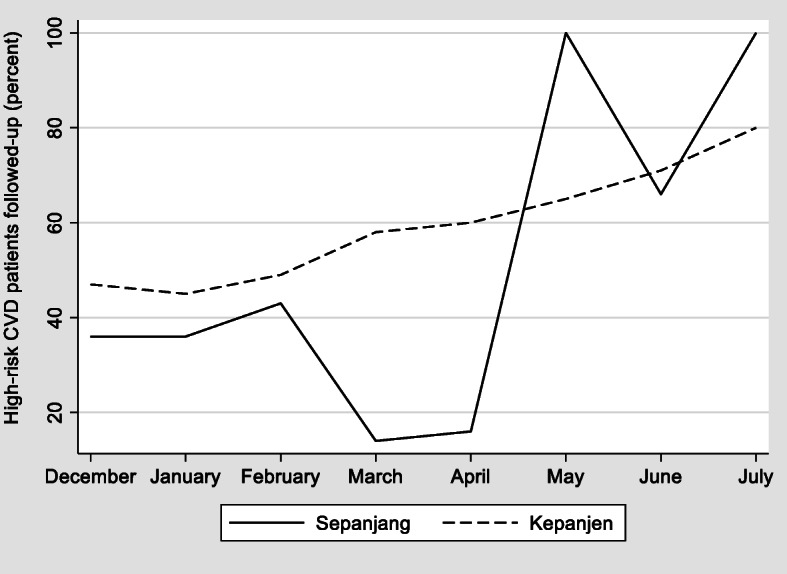
Proportion of high-risk CVD patients in each study village actively followed up by Kaders each month

In the financial incentive village (Sepanjang), Kader follow-up rates were affected by a number of politico-social and health system influences at the village level, which were outside of the control of the study (see Fig. 1). These barriers and facilitators were collected as part of the evaluation to give context to the incentive outcomes. Key barriers and facilitators to successful patient follow-up emerging from the focus group discussions with Kaders and patients are summarised below:

#### Availability of nurses

From March to April, the nurse from the Village Health Post (Ponkesdes) in Sepanjang village was temporarily assigned to administrative duties. During this time, the position remained unfilled and additional barriers of distance and geography prevented most patients from accessing the only alternative, the PHC Centre (Puskesmas). As a result, patients were unable to refill their medication prescription, which had a clear negative impact on the follow-up activity of Kaders. Unable to provide full care for patients, Kaders were reportedly disillusioned and consequently reduced their frequency of follow-up. They felt that they had lost the trust of patients during this period.

#### Perceived safety

Village government elections took place in Sepanjang village in April 2019 and had a detrimental impact on Kaders’ perceived safety. As representatives of the SMART*health* programme, Kaders felt that they were used as political messengers by the contending parties, which placed them at risk of physical violence. As a result, Kaders limited their household follow-up activities in the months leading up to the village election. In contrast, the non-financial incentive village (Kepanjen) had an appointed village head and therefore did not have village elections, and Kaders did not experience any impact of political unrest.

#### Reaching male high-risk patients

Males were much less likely to receive Kader follow-up visits compared to females across both study villages. According to focus group discussions with male patients, most work during the time that Kaders conduct home visits and they perceived Posbindu activities as female-only. Additionally, males tended to be less aware of the importance of controlling CVD risk factors and took less responsibility for their health, especially when asymptomatic. Kaders in the financial incentive village had more success in reaching male high-risk patients when they attended men-only social or religious meetings; in these cases, Kaders had to be accompanied by their husbands to attend these events and conduct their follow-up tasks.

#### Supportive communication

WhatsApp was used by Kaders in both villages to communicate with their supervisor, health centre staff, their peers and village members. According to interviews with programme administrators, this was an important communication channel that provided Kaders an opportunity to discuss and problem-solve barriers to following up patients.

### Motivational survey

The mean scores for each of the three motivation survey domains (management, performance and individual) at pre- and post-implementation are presented in Table 3. In the non-financial intervention village (Kepanjen), there was a statistically significant increase in scores across each domain, with the greatest gain in the performance domain. Motivation scores in the financial intervention village (Sepanjang) increased by a smaller margin, with a statistically significant increase seen in the performance domain only. The lowest mean score (mean = 2, i.e. disagree) was recorded in Sepanjang village post-implementation for the statement ‘maintenance/supply of my equipment and consumables is prompt and reliable’, possibly reflecting the shortage in government-supplied cholesterol test strips and inability of patients to refill medication at the time of implementation.

**Table 3:** Kader motivation survey scores at pre- and post-implementation in the two study villages

Survey domain	Pre-implementation average score	Post-implementation average score	Difference	P value
**Non-financial incentive (Kepanjen village, n = 20)**				
**1: Management**	3.04	3.51	0.47	P < 0.0001
**2: Performance**	3.05	3.85	0.79	P < 0.0001
**3: Individual**	3.18	3.63	0.45	P < 0.0001
**Financial incentive (Sepanjang village, n = 20)**				
**1: Management**	3.15	3.29	0.14	P = 0.0329
**2: Performance**	3.17	3.48	0.31	P < 0.0001
**3: Individual**	3.18	3.2	0.02	P = 0.2585

### Preliminary effectiveness

The 40 participating Kaders undertook 4084 patient follow-up visits over the 6-month study. The average patient follow-up rate was 64% (376/587 high-risk patients) in the non-financial incentive village and 56% (317/566 high-risk patients) in the financial incentive village. Figure 1 presents the proportion of high-risk CVD patients in each village actively followed up at least once by Kaders each month, including the 2 months (December 2018 to January 2019) prior to commencement of the 6-month feasibility study. Compared to pre-implementation, the average monthly follow-up rate over the 6-month study period was 17% higher among Kaders eligible for the non-financial incentive and 21% higher among Kaders eligible for the financial incentive.

## Discussion

To our knowledge, this is the first study to assess the feasibility and acceptability of a financial and non-financial incentive for CHWs in Indonesia. Our findings suggest that both types of incentives were feasible and acceptable to Kaders and project administrators, yet there was a slightly stronger preference for financial incentives among Kaders. Furthermore, both types of incentives appeared to have a positive impact on Kader service delivery outcomes and marginally increased motivational survey scores over the 6-month implementation period, despite implementation being interrupted by contextual factors outside of the control of the study.

This feasibility study shows that performance-based financial and non-financial incentives are broadly acceptable to the participating community health workforce in Indonesia and appropriate for further testing in a larger trial. Nevertheless, some adjustments may be required to improve sustainability and acceptability. Firstly, while Kaders expressed a greater preference for financial incentives, it is possible that this was influenced by the size of the incentive amount which, despite being informed by local wage norms and decided on by local stakeholders, was significantly (up to 20 times) greater than Kaders’ regular monthly payment. A smaller financial incentive value could be considered for a larger scale trial, with the added benefits of enhancing sustainability and policy relevance. Reduction in the value of a financial incentive would require careful deliberation, however, as low financial incentives have been found to adversely influence CHW motivation [12].

Secondly, Kaders were reportedly ambivalent about the non-financial incentive, suggesting that some refinements may be required to improve acceptability. The most important motivators identified in our qualitative research were the altruistic value and esteem gained from volunteering as Kaders. Future trials of incentives should explore how these factors may be incorporated into their design while taking care to leverage, not undermine, them. Lastly, even though we found a larger improvement in motivational survey scores among Kaders receiving the non-financial incentive than those receiving the financial incentive, it is possible that motivation was influenced by factors outside of the study. Similarly, it is possible that the motivation scores of those who received the financial incentive were influenced by contextual factors that interrupted implementation. Careful evaluation of such factors through, for example, embedding realist evaluation within a larger scale study of effect and sustainability is recommended [30].

Our findings align with an increasing evidence base reinforcing the importance of financial and non-financial incentives in motivating CHWs [8, 12, 13, 31]. Nevertheless, in the context of Indonesia, our findings remain novel. The Kader programme is one of the largest and longest-standing CHW programmes globally, yet has been subject to only limited research [24]. Furthermore, there appears to be scope for ongoing strengthening of the programme, as indicated by recent evidence demonstrating that relatively small modifications in Kader training and community engagement methods increased screening rates and motivation [32–34]. Our incentive strategy, particularly the more cost-effective and sustainable non-financial incentive, may be of interest to local policy and decision-makers as another option to enhance Kader performance, job satisfaction and retention.

Although conducted in just two villages, our study highlights the strong influence that unforeseen contextual factors can have on the implementation of community-based health systems research. In the financial incentive village, the risk of violence due to village elections and the reassignment of nurse to administrative duties led to a perceived lack of safety for Kaders and patients no longer having easy access to medication. Although these factors were outside the control of the study, they can be viewed as a reminder of the complexity of embedded implementation research and the need to consider the multiple, at times competing, demands placed on community members, and pre-existing power relations [17]. Experiences from India and Pakistan highlight the risk of violence as an important influence on CHW performance and a growing concern for female CHWs in particular [15, 24]. There is a clear need for the design of any subsequent larger scale trial to ensure that a theory of change and/or framework captures these contextual factors in data collection and allows for flexibility in implementation.

We identify two main strengths of our study. Firstly, the intervention was collaboratively designed with local health system stakeholders to enhance local ownership. Secondly, our contextualised head-to-head comparison of a financial and non-financial incentive aligns with the recent WHO recommendations. We are aware of only onestudy that compares financial and non-financial incentives for CHWs, which found that non-financial incentives had a stronger impact on CHWs’ service delivery outcomes [35]. Future research is encouraged to continue this line of research and test appropriate combinations of incentives.

As our study was intended to assess the feasibility and acceptability of the two different forms of incentives in preparation for a larger trial, our findings limit any conclusions relating to the sustainability of incentive impact on both Kader behaviour and health system resources. A longer period of implementation and follow-up will be necessary to assess these factors. Additionally, the inherent limitations of self-report measures should be noted when considering the findings of the motivational survey; it is possible that these were affected by response bias, with participants exaggerating improved motivation constructs.

## Conclusion

It was feasible to deliver both a performance-based financial and non-financial incentive to Kaders in Malang District, Indonesia, and both incentive types were acceptable to Kaders and programme administrators. Evidence of preliminary effectiveness also suggests that both the financial and non-financial incentives were associated with improved motivation and service delivery outcomes. We found that contextual factors outside of the control of the study significantly impacted Kader activity levels at different times during feasibility testing, overpowering any effect of the incentive intervention. These findings will inform the next phase of incentive design, in which incentive feasibility and preliminary effectiveness will need to be considered alongside their longer-term sustainability within the health system. Findings also reinforce the importance of articulating a theory of change and mechanisms to both assess and account for context (e.g. embedded process and/or realist evaluation) within the design of any subsequent larger scale trial.

## Supplementary Information

**Additional file 1:.** Motivation Measurement Tool (adapted from Prytherch 2012)

**Additional file 2:.** The TIDieR (Template for Intervention Description and Replication) Checklist*. Information to include when describing an intervention and the location of the information

## Data Availability

The data generated and/or analysed during this study are available from the corresponding author upon reasonable request.

## Abbreviations

CHW: Community health worker
CVD: cardiovascular disease
IDR: Indonesian rupiah
LMIC: Low- and middle-income country
USD: United States dollar
WHO: World Health Organization
