# Supplementary material for: Assessing the feasibility and acceptability of a financial versus behavioural incentive-based intervention for community health workers in rural Indonesia
Source: Pilot Feasibility Stud. 2021 Jun 23;7:132. doi: 10.1186/s40814-021-00871-7 (PMC8220784; doi:10.1186/s40814-021-00871-7)
Supplement: Supplementary file 1 — Additional file 1:. Motivation Measurement Tool (adapted from Prytherch 2012) [file 40814_2021_871_MOESM1_ESM.docx]

**Additional File 1: Motivation Measurement Tool (adapted from Prytherch 2012)**

| **Construct** | **Q** | **Section** |
| --- | --- | --- |
|  |  | **Management Aspects** |
| Work organization | 1 | This facility provides everything I need to perform well at work |
|  | 2 | There are enough health providers to do the work in this facility |
|  | 3 | Too often the referral system does not work efficiently |
|  | 4 | Maintenance of broken equipment at this facility is prompt and reliable |
| Competence strengthening | 5 | My job duties and responsibilities are clear and specific |
|  | 6 | Relevant guidelines are easy to access at this facility |
|  | 7 | I often feel left alone when I have to make difficult decisions about a patient’s care |
|  | 8 | I regularly have access to relevant trainings to keep my skills up to date |
| Role of performance | 9 | My performance is appraised regularly |
|  | 10 | Promotions do not depend on how well or badly one works on the job |
| Self-efficacy | 11 | It is difficult for me to speak openly to my superiors about how things are really going at work |
|  | 12 | Suggestions made by health workers on how to improve the facility are generally ignored |
| Provider feels valued/exploited | 13 | The facility management shows very little concern for me |
|  | 14 | Our rights as health workers are generally not respected |
|  |  | **Performance Aspects** |
| Competence strengthening | 1 | I do not get feedback from my superiors so it is hard to improve my performance |
|  | 2 | The feedback I get from my co-workers helps me to improve my work |
| Role of performance | 3 | Good performance is recognized by our superiors |
|  | 4 | This facility has a fair system for rewarding staff |
|  | 5 | Some of the team members work well, yet others do not and so this facility doesn’t perform well overall |
|  | 6 | We do not know how our facility is performing compared to others in the district |
|  | 7 | Our facility has clear goals that we are working towards |
|  | 8 | I am keen use any new tools to improve my performance |
|  | 9 | This facility has a good reputation in the community |
| Meaningfulness | 10 | I understand how my work contributes to the facility’s overall goals |
| Attitudes to patients | 11 | It makes me feel appreciated when patients are grateful |
| Pride/shame | 12 | I am proud to be working for this health facility |
|  | 13 | I am proud to tell others that I work in maternal and neonatal health care |
|  |  | **Individual Aspects** |
| Self-efficacy | 1 | I usually cope well with changes at work |
| Commitment | 2 | I intend to leave this facility as soon as I can find another position |
|  | 3 | I would recommend to my children that they choose a profession in maternal health care |
|  | 4 | I am willing to put in a great deal of effort to make this facility successful |
| General & intrinsic motivation | 5 | These days I feel motivated to work as hard as I can |
|  | 6 | My profession helps me to achieve my goals in life |
| Job satisfaction | 7 | Overall, I am very satisfied with my work in maternal and neonatal care |
|  | 8 | I am very satisfied to have a position where one works closely with the community |
|  | 9 | This job gives me a feeling of achievement and accomplishment |
| Timeliness and attendance | 10 | I am punctual about coming to work |
|  | 11 | I work hard to make sure that no patient has to wait a long time before being seen |
| Conscientiousness | 12 | I am careful not to make errors at work |
|  | 13 | When I am not sure how to treat a patient’s condition I look for information or ask for advice |
| Cooperativeness | 14 | I try to get on well with the other health staff because it makes the work run more smoothly |
|  | 15 | I get along well with my superiors at work |
